# Experience of people with Biochemical Genetic Disorders and their families accessing Genetic Counselling and Genetic Testing in the Irish Republic

**DOI:** 10.1007/s12687-025-00791-6

**Published:** 2025-04-02

**Authors:** Arnott C, Ward AJ, Lambert DM, Butterly D, McGrath V, Lynch SA, O.’Byrne JJ

**Affiliations:** 1 School of Medicine, University College Dublin, Dublin, Ireland; 2 Rare Diseases Ireland, Dublin, Ireland; 3 Clinical Genetics, Children’s Health Ireland (CHI) at Crumlin, Dublin, Ireland; 4 Mater Misericordiae University Hospital (MMUH), National Centre for Inherited Metabolic Disorders, Dublin, Ireland; 5 School of Medicine, Trinity College Dublin, Dublin, Ireland

**Keywords:** Genetic counselling, Genetic testing, Patient experience, Rare metabolic disease, Background

## Abstract

**Background:**

National and international reports recommend that genetic counselling should be made available to parents of children living with inherited rare diseases; and to patients themselves upon turning 16–18 years old. Long wait times of up to two years for genetic counselling through Children’s Health Ireland contributed to a lack of accessibility for adult patients with inherited metabolic disorders (IMDs). At the time of the study, the National Centre for Inherited Metabolic Disorders (NCIMD) Mater, which takes care of ~ 1400 adult patients with genetic disorders primarily affecting biochemical pathways, did not have direct access to a genetic counsellor.

**Objectives and methods:**

An online survey was conducted to investigate the genetic testing and counselling experiences of adult patients with rare IMDs and their families within the Republic of Ireland.

**Results:**

The NCIMD-Mater survey highlighted a lack of patient knowledge of and access to genetic counselling services; with some patients unaware of and others incorrectly understanding the role of genetic counselling. Most patients who underwent genetic testing were tested by a non-genetic healthcare professional. Satisfaction levels of genetic counselling services were mixed with some patients reporting delaying personal life and family plans due to wait times for genetic counselling.

**Conclusion:**

This study highlights deficiencies in the genetic testing and counselling experience of Irish IMD patients. Embedding genetic counselling into multidisciplinary IMD teams would increase access to genetics education for patients and families and improve the clinical service. This study may be utilized to measure the impact of integrating genetic counsellors into NCIMD-Mater.

**Supplementary Information:**

The online version contains supplementary material available at 10.1007/s12687-025-00791-6.

National and international reports on the transition of healthcare for young people living with rare diseases recommend that young people living with rare diseases transitioning into adult healthcare systems should be informed of the genetic counselling service at the age of 16–18 years old (O’Shea et al. 2011; Taylor et al. 2006). The Health Service Executive Model of Care for Rare Diseases (2019) highlights the necessity for genetic testing, preceded and followed up by genetic counselling, based on recommendations from European studies (Matthijs et al. 2016). The Irish Health Service Executive National Strategy for Accelerating Genetic and Genomic Medicine in Ireland (2022) strives to strengthen local access to genetic counselling with a commitment for funding in 2023.

Genetic counsellors are tasked with providing information, counselling and support to patients living with genetic conditions and their families, or to those who may be at risk of these conditions (Snider et al. 2020). Knowledge imparted to patients by genetic counsellors can contribute to informed decision-making capacity for treatment and reproductive decisions (Conway et al. 1996) and is important for patient satisfaction and compliance (Stein et al. 2017). According to the National Treatment Purchase Fund in January 2022, there were 4,122 patients awaiting clinical genetics appointments at the Clinical Genetics Department of Children’s Health Ireland in the Republic of Ireland, with 952 having been on the waiting list for over 18 months. Whilst these are Consultant waiting lists only, Genetic Counsellor waiting lists are similar (personal communication SA Lynch). Chronic staff shortages of clinical staff, both genetic counsellors and consultant clinical geneticists, are a recognized driver of the waiting list.

In the Republic of Ireland, all patients with Inherited Metabolic Diseases are followed by one of two tertiary multidisciplinary departments: children at the National Centre for Inherited Metabolic Disorders at Children’s Health Ireland and from age 16 at the National Centre for Inherited Metabolic Disorders at the Mater Misericordiae University Hospital (NCIMD-Mater). While some patients may also have day to day care in collaboration with their regional hospital, the NCIMD oversees their care and is the only service that offers care to patients who are planning pregnancy or are pregnant. Approximately 1400 patients attend, with essentially complete national ascertainment of all adults with inherited metabolic disease continuing follow-up into adulthood. Whilst genetic testing is now typically offered at diagnosis, often via the paediatric metabolic service, up until recently the adult service NCIMD-Mater team, arranged genotyping and consent was taken by the consultant clinical geneticist/metabolic specialist or junior doctor on the team (usually in rotation for 6 months).

It had previously been demonstrated (O’Shea et al. 2011) that young adults with galactosemia and MSUD attending the paediatic NCIMD (as no adult clinic was then available) did not know about the genetic basis of their condition and their risks as adults despite regular clinic attendance since early childhood. O’Shea’s study showed that these adolescents had a significant knowledge gap which may be addressed by access to genetic counselling at transition. Adolescents living with PKU have previously reported a preference for earlier access to genetic information, at the age of 12 years old (Szybowska et al. 2007); as this would involve patients earlier in understanding their condition and not relying solely on parents relaying information. During our study period, NCIMD-Mater did not have a genetic counsellor on their team and were reliant on referring to Clinical Genetics services at Children’s Health Ireland with a long waiting time.

Inherited metabolic disorders (IMDs) collectively make up a substantial group of rare genetic conditions that primarily affect biochemical pathways (1/2500–5000 live births worldwide) (Scriver et al. 2001) – with a higher prevalence in Ireland (Morrissey et al 2013). The metabolic field has previously been suggested to be an ideal field to measure the effectiveness of genetic counselling due to an identifiable biochemical pathway, autosomal recessive mode of inheritance and thus opportunity to discuss recurrence risks and effective genotype-specific treatment options in many cases (Stein et al. 2017). Stein et al. reported that genetic counselling is delivered / provided in one third of cases by other healthcare professionals including physicians, nurses and dietitians and recommended that all metabolic units should have a genetic counsellor embedded in their multidisciplinary team (MDT). Do et al. (2024) emphasize the ongoing transition of genetic counselling from clinical genetics services to wider models of care and mainstream clinics, as genetic counsellors’ roles expand with genomic healthcare demands. Within the past 25 years the treatment of metabolic diseases has allowed those affected to survive and thrive in adulthood and consider their own reproductive options. More recently, variant-based treatment of inherited metabolic conditions increases the need for genetic testing to direct therapeutic treatment. For both these reasons, metabolic clinics are a logical opportunity for mainstreamed genetic counsellors. The primary aim of this study was to establish a baseline of knowledge about genetic testing and genetic counselling in patients of reproductive age in the NCIMD-Mater, in order to inform future practice.

## Methods

In this study a group of adult patients with IMDs attending the NCIMD-Mater were surveyed to investigate their experience of genetic testing and genetic counselling in the absence of genetic counsellor within the service.

### Survey development

An online survey was developed based on our previous survey of members of the public accessing or waiting to access genetic testing or genetic counselling in the Republic of Ireland (Ward et al 2023). This public survey had been co-written with Rare Diseases Ireland, the national rare diseases patient advocacy group. Questions for the public survey were drawn from Eurordis Rare Barometer surveys n.d. and adapted for local use. For this purposive metabolic survey we adapted questions from the previous survey (Ward et al 2023) to reflect that participants had, rather than were seeking, a genetic diagnosis. We used responses to the previous survey (Ward et al 2023) to judge coherence of the wording of questions to be included in the current survey. The survey was written in accordance with quality guidelines for Internet surveys of the CHERRIES quality checklist (Eysenbac 2004). Survey logic was applied to gatekeeping questions within the online survey to direct respondents to relevant sections.

Approval for this survey was obtained from the MMUH Research Ethics committee (IRB Reference No: 1/378/2275). The data protection officer at MMUH approved the completed data protection impact assessment. The survey (excluding consent questions) consisted of 25 tick-box questions pertaining to patient demographics, genetic testing, genetic counselling and the impact of waitlists. Survey respondents were asked to describe their experience of genetic testing and genetic counselling in their own words in free text boxes within these two sections.

### Study participants

NCIMD-Mater patients with a diagnosis of a rare metabolic condition who were known to be planning a pregnancy, pregnant or within 12 months postpartum, and their partners were identified by the NCIMD-Mater clinical team as the target survey group. All participants were required to be at least 18 years of age upon survey completion.

### Participant recruitment

NCIMD-Mater clinical staff invited 47 patients and their partners to participate in this study by email with a follow-up supportive information phone call. Participants were informed at the time of the call that the survey was anonymous and would not influence their clinical care. The online survey was open to responses from 12th September 2022 to 24th October 2022.

### Data cleaning and analysis

A completion check of submitted responses was conducted, removing those with a low completion level (less than 50%) and those completed in under 2 min, as recommended by the CHERRIES quality checklist (Eysenbach G, 2004). Duplicate responses were removed via IP analysis (with the exception of cases where the patient and a partner utilised the same device to respond). Data cleaning was carried out to redact potentially identifiable information. Quotes were redacted if permission was not given for their usage.

Data was analysed with Survey Monkey and Excel. Simple descriptive statistics were calculated for each question. Small sample size (despite complete national ascertainment) did not permit either stratified analysis or tests of statistical significance. Thematic analysis was not possible as data saturation was not reached due to the brevity and small number of quotes received. Quotes are presented for illustrative purposes.

## Results

### Respondents

Of the 47 patients contacted, 31 eligible survey responses were received, of which 97% (30/31) were patients of the NCIMD-Mater and 3% (1/31) partners. Phenylketonuria (PKU) accounted for 87% (27/31) of diagnoses. The majority of respondents completed the questionnaire in their first year after giving birth, with most other respondents planning a pregnancy or pregnant upon survey completion. Ninety-one per cent (28/31) of participants were 26–45 years old at the time of survey completion. The age of diagnosis for 87% (27/31) was < 1 year, 26/27 were PKU diagnoses. Table 1 displays the full demographic information of survey respondents.

**Table 1:** Demographic characteristics of respondents completing the survey

*Table 1 Demographics*		N	%
*Gender (n* = *31)*	Female	30	97%
	Male	1	3%
*Ethnicity (n* = *31)*	Irish	30	97%
	Other White	1	3%
*Age category (n* = *31)*	18–25	1	3%
	26–35	16	52%
	36–45	12	39%
	45 +	1	3%
*Family diagnosis (n* = *31)*	PKU	27	87%
	Other	4	13%
*Pregnancy Status(n* = *31)*	Planning/considering pregnancy	7	23%
	Pregnant	5	16%
	Post-delivery (< 1 yr)	16	52%
	Not planning/considering pregnancy	3	10%
*Age of patient/family diagnosis (n* = *31)*	< 1 yr	27	87%
	1–5 years	1	3%
	6–15 years	1	3%
	16–25 years	1	3%
	26–50 years	1	3%

### Experience of genetic testing

**Fig. 1:**
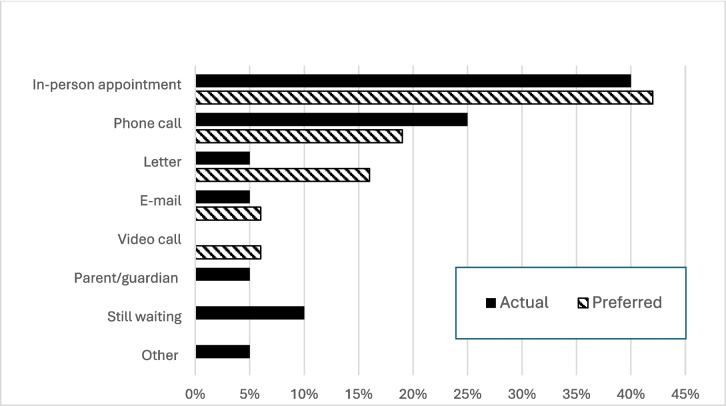
Methods of genetic testing results delivery, actual (n = 20) vs preferred (n = 31). Participants were given the option to tick all applicable options

Of note, 65% (20/31) of the survey respondents reported having had genetic testing, with a third of the remaining participants (4/11) unsure whether they had undergone genetic testing. Only 20% (4/20) of genetic tests were arranged by a Consultant Clinical Geneticist, with 0% (0/20) of tests arranged by genetic counsellors. Five out of twenty respondents who underwent genetic testing reported receiving results from a different healthcare worker to their initial referrer, including receiving results from nurses and during general metabolic unit appointments. Waiting times for genetic testing results exceeded one year in 20% (4/20) of cases, reflecting the fact that many of these tests were conducted sometime in the past, with 10% (2/20) still awaiting results at the time of the survey. The majority of patients received their genetic testing results by direct human interaction, either via in-person appointment (44%, 8/18) or over the phone (28%, 5/18). Notably, in-person appointments (42%, 13/31) and phone calls (19%, 6/31) were also preferred for genetic test result communication as shown in Fig. 1. Complete univariate analysis of genetic testing question responses is contained in Table 2. Free text quotes on the topics of genetic testing are presented in Table 3. Emerging themes include: long waiting times for appointments and results availability; a lack of recommendation for genetic counselling and resultant uncertainty.

**Table 2:** Univariate results: genetic testing in survey

		N	%
*Genetic testing? (n* = *31)*	Yes	20	65%
	No	7	23%
	Not sure	4	13%
*Wait time for genetic testing results (n* = *15)*	0–3 months	3	15%
	4–6 months	1	5%
	7–12 months	2	10%
	13–18 months	1	5%
	> 18 months	3	15%
	Unsure	3	15%
	Still waiting	2	10%

**Table 3:** Emerging themes from free-text quotes about genetic testing

THEME		n	Quote example
1	Highlighted long waiting times for appointments and results availability	4	‘On one of my appointments in the Metabolic unit I was told I could test to see if I was eligible for Kuvan®. They did the bloods and never sent them to be tested. Another appointment 6 months later or possibly more they did bloods again and these too were not tested. I just stopped asking then. This year out of the blue in my appointment they told me they had the results and I would be a candidate for Kuvan®…’ ‘Communication can be active when seeking volunteer for research or testing but getting feedback is slow and difficult.’ ‘By the time I got the results of the genetic test I had completely forgotten I had got it done. Also, all the genetic testing did for me was to confirm that I had PKU … I had known that my whole life.’ ‘I had to ring up myself to find out the result …’
2	Indicated a lack of recommendation for genetic counselling and resultant uncertainty	3	‘After receiving results of genetic testing I inquired about genetic testing for my partner and possible genetic counselling but was dismissed as unnecessary by metabolic consultant in the Mater.’ ‘I have never been offered genetic counselling and not sure what this involves.’ ‘I got my blood test taken on a routine check up. I had to ring up myself to find out the result and only got a brief result. I would’ve liked more information and details on how to move forward or if what options were available.’
3	Highlighted a role for virtual appointments, especially for high-risk individuals during, for example, the COVID-19 pandemic	1	‘I did not attend appointments during covid19 pandemic as I was in the high-risk category, so I felt the option of virtual appointments could have been offered instead as it was quite a break between appointments for a couple of years and I was at a stage of family planning.’

### Experience of genetic counselling

**Fig. 2:**
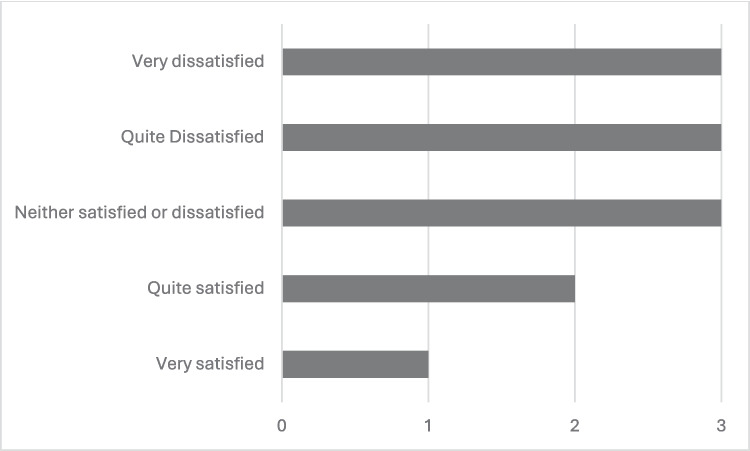
Satisfaction levels of patients attending genetic counselling (n = 12)

Overall, 97% (30/31) reported getting information about their condition from the metabolic service, 35% (11/31) from family members and 29% (9/31) from the internet. With regards to genetic counselling, 55% (17/31) reported being unaware of the option of genetic counselling and 19% (6/31) were unsure if they had ever had genetic counselling from either the paediatric or adult services. Thirteen per cent (4/31) of participants met with a genetic counsellor in childhood, with only 10% (3/31) of respondents having met with a genetic counsellor in adulthood. Of note, 52% (13/25) of respondents reported that they had not been referred to the genetic counselling service but had a desire to be referred. Of the 16% (4/25) of participants who had already been referred to and attended a genetic counsellor appointment, 75% (3/4) attended a public appointment. Overall satisfaction varied among participants, as displayed in Fig. 2. Table 4 shows further univariate data pertaining to the genetic counselling experience.

**Table 4:** Univariate results: genetic counselling questions

		*N*	%
*Genetic counselling contact (n* = *31)*	Met with a genetic counsellor in adulthood	3	10%
	Met with a genetic counsellor in childhood	4	13%
	Awaiting genetic counselling	1	3%
	Unaware of the option of genetic counselling	17	55%
	Not sure	6	19%
*Source of information about condition (Tick all that apply) (n* = *31)*	Family members	11	35%
	Metabolic team	30	97%
	Genetic team	1	3%
	Internet	9	29%
	Patient organization	6	19%
Referral status to Genetic Counselling (n = 25)	Yes—have already attended	4	16%
	Yes—waiting for first appointment	0	0%
	No—have not been referred, but would like to be	13	52%
	No—have not been referred and do not want to be	5	20%
	Not sure	3	12%
Type of genetic counselling appointment or referral (n = 4)	Private	0	0%
	Public	3	75%
	Not sure	1	25%
Wait time for genetic counsellor appointment (n = 4)	0–3 months	3	75%
	3–6 months	0	0%
	6–12 months	0	0%
	12–15 months	0	0%
	> 15 months	0	0%
	> 15 months	1	25%

Eight participants gave permission to share quotes from their responses to the open-ended question about their experience of genetic counselling. Key themes included: a need for more information regarding the availability and benefits of genetic counselling, a wish for access to genetic counselling in pregnancy planning for patients and their partners, including carrier testing and positive experience of genetic counselling. Respondents’ full quotes are outlined in Table 5.

**Table 5:** Emerging themes from free-text quotes about genetic counselling

THEME		n	Quote example
1	Highlighted a requirement for more information regarding the availability and benefits of genetic counselling	3	‘I'm not sure if I have been given any information on this or been informed from my team in the Mater.’ ‘More information should be available about genetic counselling.’ ‘I wasn’t given any information about the options or reasons for genetic counselling.’
2	Expressed a positive experience of genetic counselling	3	‘I have had genetic counselling from the metabolic team explaining how the gene is passed on, the likelihood of children having PKU in general. I feel this was absolutely sufficient for me.’ ‘It was very informative and give me a good insight into what would happen after I had children and the chances of them having the condition. It gave peace of mind.’ ’ I found the service very informative … I will keep in contact throughout my pregnancy and into the future to make sure I am well informed for the sake of my child.’
3	Expressed a wish for a genetic counselling role in pregnancy planning for patients and their partners, including carrier testing	2	‘It would be beneficial to patients planning pregnancy to have genetic counseling available to them and their partners.’ ‘It would be nice to have my partner tested to know if he was a carrier of the PKU gene or not …’
4	Expressed concern about the impact of long waitlists	1	‘It would be beneficial to patients planning pregnancy to have genetic counselling available to them and their partners. In such situations, long waiting lists would be problematic.’
5	Highlighted the importance of remote appointments for patients	1	‘as I live quite a distance from Dublin, the ease of having it over the phone was brilliant.’

### Impact of waiting list

Results to questions regarding the impact of waiting lists are included in Table 6. One participant underwent genetic testing with a public consultant while awaiting their genetic counselling appointment. Three of the thirty-one participants underwent genetic testing, via a clinical research study. There were very few responses to questions about the effect of waiting times to see a genetic counsellor. Six per cent (2/31) of patients reported that delays in access to genetic counselling caused a delay in marriage / settling down or starting a family.

**Table 6:** Impact of waiting list on patients

		*N*	*%*
While waiting for genetic counselling in Ireland	Had genetic testing via GP / family doctor	0	0%
	Genetic testing via public consultant	1	3%
	Private genetic testing in Ireland	0	0%
	Private genetic testing or appointment is unaffordable	1	3%
	Appointment to see a Genetic Consultant or Genetic Counsellor via the Cross-Border Directive or the Treatment Abroad Scheme	0	0%
	Genetic testing via research study / clinical trial	3	9%
Reported impact of waiting time on personal life	Delayed plans to have more children	0	0%
	Delayed plan to marry/settle down/commit to a relationship	1	3%
	Delayed plans to start a family	1	3%
	Delayed plans for mortgage or insurance	0	0%
	Changed/delayed employment	0	0%
	Changed/delayed education	0	0%
	Placed tension on relationships with partner, family members or friends	0	0%
	Wider impact on relative's family planning/ relationships/ education/ employment plans	0	0%

### Genetic counsellor role

**Fig. 3:**
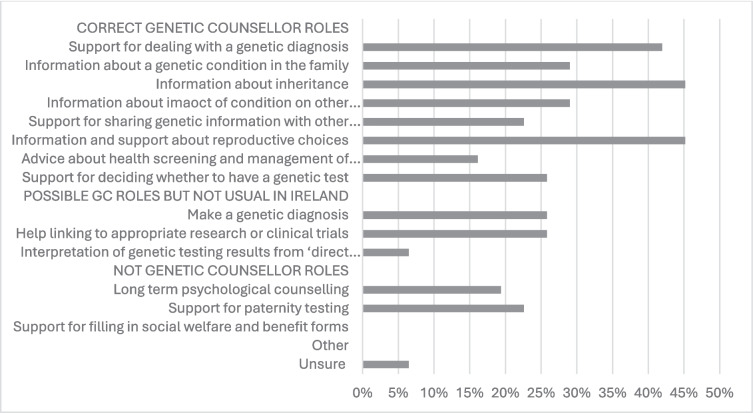
Participant (n = 31) responses as to what they believe is included in the role of genetic counsellor

Respondents were asked to recognize the role of genetic counsellors from a list of possible options. Over 40% of participants correctly identified ‘for dealing with a genetic diagnosis’, ‘information about how the condition is passed on (inherited) in the family’ and ‘information and support about reproductive choices for a future pregnancy’ (42%, 45% and 45% respectively) as roles of genetic counsellors. Providing support for deciding whether to have a genetic test was amongst the lesser recognized of genetic counsellors’ roles (26%). Over a quarter of participants (26%) believed that genetic counsellors can make a genetic diagnosis and help to link the patient to appropriate research or clinical trials (see Fig. 3).

Patients who had not been referred to genetic counselling, but had a desire to be referred had a similar level of understanding as the rest of the survey cohort of the most commonly identified roles of genetic counsellors ‘for dealing with a genetic diagnosis’ and providing ‘information about how the condition is passed on (inherited) in the family’ (38% and 46% respectively), but seemed less aware of the role of genetic counsellors in provision of ‘information and support about reproductive choices for a future pregnancy’ compared to the rest of the survey respondents (31%). However, due to the limited numbers no statistical analysis was possible.

## Discussion

This survey aimed to explore the genetic counselling and testing experience of people living with IMDs in ROI and who attend NCIMD-Mater, to provide a baseline upon which to evaluate the validity of the forthcoming role for genetic counsellors embedded within the service. The subgroup of patients selected were planning pregnancy, pregnant or within 12 months postpartum, placing a particular focus on the impact of genetic counselling on pregnancy planning within the rare disease community. A high proportion, 87% (27/31), of this cohort were diagnosed in childhood including 26/27 diagnosed with phenylketonuria (PKU) by the Irish new-born screening program (Health Service Executive 2018).

In our study, 19% of survey respondents were unsure if they had previously had genetic counselling and 55% were unaware of this option. 42% of respondents reported a desire to be referred for genetic counselling. This confirms previous findings in this patient cohort that the transfer of genetic information from parents to their children affected with IMDs can be limited (O’Shea et al. 2011) and may account for the relatively low awareness of genetic counselling.

There is a need for public education about the availability of genetic counselling and the role of genetic counsellors. Over half of respondents reported a lack of awareness about genetic counselling services, which mirrors the larger public survey published by our group (Ward et al. 2023). Education on and raising awareness of genetic counselling is planned as part of the National Strategy for Accelerating Genetic and Genomic Medicine in Ireland (Health Service Executive (2022).

Survey findings highlight that adults with IMDs would value an opportunity to access genetic counselling to explore their genetic testing results and clarify reproductive risks. With wait lists of up to two years for genetic services in Ireland (Bradley and Lynch 2021), over 2,799 waiting for Consultant Clinical Geneticist outpatient appointments in December 2022 (National Treatment Purchase Fund 2022) and similar Genetic Counsellor waiting lists (personal communication SA Lynch); the Irish Health Service Executive (2019) acknowledges such delays as an issue. Two participants in this study reported that wait times for genetic counselling appointments had a direct impact on their personal lives by delaying relationship and family-starting plans.

This survey suggests that patients value human interaction when receiving genetic testing results as in-person, telephone or video call were preferred methods of communication. However, comments in the free-text boxes revealed a role for telehealth as an alternative to in-person appointments to improve access for patients living remotely. The positive role for telehealth in genetic counselling has previously been reported, Abrams and Geier (2006), who noted adequate patient satisfaction.

Although NCIMD-Mater has a clinical geneticist on staff, responses to our survey still indicate a desire for genetic counselling referral. This echoes findings by Bartley et al (2020) where participants in an oncology study stated that they preferred to have a genetic counsellor rather than a medical geneticist or oncologist explain genomic testing results, also placing emphasis upon the importance of communication skills.

Development of diagnostics and therapeutics has improved general management for patients with IMDs, allowing lengthening of lifespans and the preservation of intellect in many. Patients with these conditions are now more likely to establish relationships allowing couples to consider reproductive options. Providing information about inheritance and supports available for reproduction were the two most correctly identified roles of genetic counsellors amongst this cohort of participants (45% each, 14/31). Discussion of reproductive plans, recurrence risks and prenatal diagnosis contributes to an increased sense of personal control amongst patients regarding their future (Pichini et al. 2016) and an improved capacity for reproductive decision making (Conway et al. 1996). The pre-pregnancy genetic counselling also contributes to the discussion with mothers diagnosed with PKU, around appropriate nutrition (Hartley et al. 2011) to prevent intellectual disability, microcephaly, and congenital heart disease (CHD) resulting from untreated maternal PKU (Prick et al. 2012).

The advent of genotype-specific treatments in the field of metabolic medicine such as sapropterin, Kuvan®,(tetrahydrobiopterin) for PKU or migalastat for Fabry Disease increases the drive for genetic testing in precision medicine. Stein et al. 2017, recommend that all metabolic units have genetic counsellors embedded in their MDTs for genetic informational and support purposes, especially as rising numbers of patients opt for genetic testing. Additionally, Hartley et al. (2011) note that metabolic clinics’ structure would aid effective genetic counselling practices by enabling a longitudinal relationship to build between genetic counsellors and their patients. This survey represents a baseline to assess the impact of the integration of a genetic counsellor into the metabolic service. Since the study was conducted, a genetic counsellor has been recruited to the team to improve the awareness of genetic testing amongst the patients and the implications of their genetic diagnosis within routine metabolic clinic appointments.

### Limitations of the study

This survey appreciated low response rates from male patients and partners (3.2%, 1/31) and from people of a non-Irish white background (3%, 1/31). There were no respondents from a Black, Asian, or mixed ethnic background. Due to the limited respondent numbers statistical analysis was not possible.

## Conclusion

At the time of the survey, access to genetic counselling for Irish adults with IMDs required referral to an external service with an extensive waiting list. The respondents in this study indicated a desire for access to genetic counselling services. There was a lack of knowledge around genetic testing in a cohort of patients who can benefit from genotype-specific for precision medicine therapies. The multidisciplinary structure of metabolic healthcare provision, with strong emphasis on regular follow-up, provides the ideal opportunity for building a longitudinal relationship between genetic counsellors and their patients. This study provides a baseline to allow the impact of embedding a genetic counsellor in metabolic teams to be measured. It also contributes to proof of concept in favour of the provision of genetic counselling services within metabolic clinics.

## Supplementary Information

Below is the link to the electronic supplementary material.

Supplementary file1 (PDF 94 KB)

## Data Availability

No datasets were generated or analysed during the current study.

## References

[CR1] Abrams DJ, Geier MR (2006) A comparison of patient satisfaction with telehealth and on-site consultations: A pilot study for prenatal genetic counseling. J Genet Couns 15(3):199–205. 10.1007/s10897-006-9020-0 16779676 10.1007/s10897-006-9020-0

[CR2] Bartley N, Napier C, Best M, Butow P (2020) Patient experience of uncertainty in cancer genomics: a systematic review. Genet Med 22(9):1450–1460. 10.1038/s41436-020-0829-y 32424175 10.1038/s41436-020-0829-y PMC7462749

[CR3] Bradley L, Lynch SA (2021) Dying to see you? Deaths on a clinical genetic waiting list in the Republic of Ireland; what are the consequences? J Community Genet 12(1):121–127. 10.1007/s12687-020-00491-3 33119819 10.1007/s12687-020-00491-3 PMC7846633

[CR4] Conway SP, Pond MN, Watson A, Hamnett T (1996) Knowledge of adult patients with cystic fibrosis about their illness. Thorax 51(1):34–38. 10.1136/thx.51.1.34 8658366 10.1136/thx.51.1.34 PMC472796

[CR5] Do TT, Martyn M, McClaren B, McEwan A, Gaff C (2024) Becoming agents for genomic change: genetic counsellors’ views of patient care and implementation influences when genomics is mainstreamed. Eur J Hum Genet. 10.1038/s41431-024-01686-9 39210048 10.1038/s41431-024-01686-9 PMC11606944

[CR6] Eurordis Rare Barometer Surveys https://www.eurordis.org/resources-search/?keywordSearch=&filterPostType=publications&filterPublicationsTag=rare-barometer-survey-results&daterange= Accessed 05 August 2024

[CR7] Eysenbach G (2004) Improving the quality of Web surveys: the Checklist for Reporting Results of Internet E-Surveys (CHERRIES). J Med Internet Res 29;6(3):e34. 10.2196/jmir.6.3.e34 Erratum in: 10.2196/jmir.2042 10.2196/jmir.6.3.e34 PMC1550605 15471760

[CR8] Hartley JN, Greenberg CR, Mhanni AA (2011) Genetic counseling in a busy pediatric metabolic practice. J Genet Couns 20(1):20–22. 10.1007/s10897-010-9324-y 20839038 10.1007/s10897-010-9324-y

[CR9] Health Service Executive (2018) A Practical Guide to Newborn Bloodspot Screening in Ireland (7th edition). https://www.hse.ie/eng/health/child/newbornscreening/newbornbloodspotscreening/information-for-professionals/a-practical-guide-to-newborn-bloodspot-screening-in-ireland.pdf Accessed 05 August 2024

[CR10] Health Service Executive (2019) Model of care for rare diseases. https://www.lenus.ie/handle/10147/626904 Accessed 05 August 2024

[CR11] Health Service Executive (2022) National strategy for accelerating genetic and genomic medicine in Ireland. In: National Strategy for Accelerating Genetic and Genomic Medicine in Ireland. https://www.hse.ie/eng/about/who/strategic-programmes-office-overview/national-strategy-for-accelerating-genetic-and-genomic-medicine-in-ireland/national-strategy-for-accelerating-genetic-and-genomic-medicine-in-ireland.pdf Accessed 05 August 2024

[CR12] Matthijs G, Souche E, Alders M et al (2016) Guidelines for diagnostic next-generation sequencing. Eur J Hum Genet 24:2–5. 10.1038/ejhg.2015.226 26508566 10.1038/ejhg.2015.226 PMC4795226

[CR13] Morrissey L, Tiernan CA, Lambert D et al (2013) Hereditary metabolic diseases (HMDs) in adult practice in Ireland: a preliminary assessment. Ir J Med Sci 182:565–571. 10.1007/s11845-013-0927-9 23526233 10.1007/s11845-013-0927-9

[CR14] National Treatment Purchase Fund (2022). https://www.ntpf.ie/home/outpatient.htm Accessed 05 September 2024

[CR15] O’Shea R, Murphy AM, Treacy E, Lynch SA, Thirlaway K, Lambert D (2011) Communication of genetic information by other health professionals: the role of the genetic counsellor in specialist clinics. J Genet Couns 20(2):192–203. 10.1007/s10897-010-9337-6 21210198 10.1007/s10897-010-9337-6

[CR16] Pichini A, Shuman C, Sappleton K, Kaufman M, Chitayat D, Babul-Hirji R (2016) Experience with genetic counseling: The adolescent perspective. J Genet Couns 25(3):583–595. 10.1007/s10897-015-9912-y 26573304 10.1007/s10897-015-9912-y

[CR17] Prick BW, Hop WC, Duvekot JJ (2012) Maternal phenylketonuria and hyperphenylalaninemia in pregnancy: pregnancy complications and neonatal sequelae in untreated and treated pregnancies. Am J Clin Nutr 95(2):374–382. 10.3945/ajcn.110.009456 22205310 10.3945/ajcn.110.009456

[CR18] Scriver CR, Sly WS, ChildsBeaudetValleKinzler BALDKW et al (2001) The metabolic and molecular bases of inherited disease. Biochem Mosc 67:611–612. 10.1023/A:1017418800320

[CR19] Snider AC, Isley LJ, Black LD (2020) Scope of practice distinctions based on primary work setting for genetic counselors in assisted reproductive technologies. F S Rep 2(1):80–87. 10.1016/j.xfre.2020.12.001 34223277 10.1016/j.xfre.2020.12.001 PMC8244313

[CR20] Stein QP, Vockley CW, Edick MJ, Zhai S, Hiner SJ, Loman RS, Davis-Keppen L, Zuck TA, Cameron CA, Berry SA, Inborn Errors of Metabolism Collaborative (2017) An Exploration of Genetic Test Utilization, Genetic Counseling, and Consanguinity within the Inborn Errors of Metabolism Collaborative (IBEMC). J Genet Couns 26(6):1238–1243. 10.1007/s10897-017-0100-0 28451876 10.1007/s10897-017-0100-0 PMC5659965

[CR21] Szybowska M, Hewson S, Antle BJ, Babul-Hirji R (2007) Assessing the informational needs of adolescents with a genetic condition: what do they want to know? J Genet Couns 16(2):201–210. 10.1007/s10897-006-9060-5 17277993 10.1007/s10897-006-9060-5

[CR22] Taylor MR, Edwards JG, Ku L (2006) Lost in transition: challenges in the expanding field of adult genetics. Am J Med Genet C Semin Med Genet 142C(4):294–303. 10.1002/ajmg.c.30105 17024669 10.1002/ajmg.c.30105

[CR23] Ward A, Lambert D, Butterly D et al (2023) Genetic services survey—experience of people with rare diseases and their families accessing genetic services in the Irish Republic. J Community Genet 14:583–592. 10.1007/s12687-023-00664-w 37632685 10.1007/s12687-023-00664-w PMC10725380

