# Supplementary material for: Experience of people with Biochemical Genetic Disorders and their families accessing Genetic Counselling and Genetic Testing in the Irish Republic
Source: J Community Genet. 2025 Apr 2;16(5):529–38. doi: 10.1007/s12687-025-00791-6 (PMC12401831; doi:10.1007/s12687-025-00791-6)
Supplement: Supplementary file 1 — Supplementary file1 (PDF 94 KB) [file 12687_2025_791_MOESM1_ESM.pdf]

Genetic services survey for patients at the National Centre for Inherited Metabolic Disorders, MMUH: Information for Participants

Genetic services survey September 2022

We would like to find out more about people's experience in Ireland of access to genetic counselling to learn about the impact of their diagnosis on themselves and their family. Please read this information to help you decide whether you would like to participate in our study.

This survey is part of a study by a research team with Dr. James O'Byrne from the National Centre for Inherited Metabolic Disorders at Mater Misericordiae University Hospital' as a co-investigator on the study. The Principal Investigator is Prof. Sally Ann Lynch based at University College Dublin and Children's Health Ireland (CHI) at Crumlin. The title of the research project is "Genetic Counselling and Testing in the Irish Republic: Scoping current practice, international comparisons and recommendations for national practice".

This survey will be open from the 13th of September to the 11th of October (now extended to the 25th October**). We will send you a gentle reminder email the week before the survey closes.**

Who can take part?
The survey should take about 15-20 minutes to complete. The National Centre for Inherited Metabolic Disorders is inviting you to take part because you are a patient aged >18 year for whom genetic counselling for your or your partner's condition would be particularly important and you live in Ireland.

What questions will the survey ask?
The survey will first check that you consent to participate in this survey. There will then be questions about:

- Your background information
- Your experience of accessing genetic counselling
- Your experiences on the waiting list for an appointment in Genetics
- Your experience of a genetic counselling appointment
- What you know about genetic counselling

Who will know I have taken part?
We will not ask for your name, address, or birth date in the survey. **The Metabolic team at the Mater Hospital will not know if you have participated.** Survey responses will be securely kept by the research team in line with data protection policy. Only the research team will have access to the survey responses, they won't be shared with anyone else.

Because we do not ask for names, we cannot identify your personal survey responses. This means if later you decide that you did not want to take part, we will not be able to withdraw your responses from the study.

How will survey responses be reported?
All the survey responses will be combined so that no single response can be identified. The research team will analyse the combined results. A report for policy makers, healthcare providers and the public will be published. We intend that the report will be available in 2022/23. You will be able to access the report at http://www.adelaide.ie/health-policy/publications-reports/ after it is published. The results of the analysis may also be reported in scientific journals and/or conferences.

Consenting to participate
It is up to you whether you choose to complete this survey. Please read this information carefully. The first survey questions will ask you if you are happy to consent to take part in the study. If you don’t want to answer the survey, that’s OK too. Choosing not to participate in the study would not affect your care in the National Centre for Inherited Metabolic Disorders at the Mater Hospital.

As part of the consent, we ask if we may quote your text answers in the study report. You may choose not to have your quotes used in the report. You can still fill out the survey if you choose ‘no’ – your answers are still helpful. If you say ‘yes’, we may use some quotes in the report. We will not use any diagnoses or any identifying information in the quotes.

However, because some situations are so rare, it is not possible to guarantee that you cannot be identified in quotes.

Privacy
Please do not use your name or the names of family members or healthcare professionals in your answers.

Who is funding the survey?
This survey work is funded by Adelaide Health Foundation (www.adelaide.ie), a voluntary independent charity which seeks to advance equal access to quality healthcare. No funders have any role or influence in design, analysis or reporting of this research.

Potential Risks and Benefits
It is very unlikely that you would have any harm from completing this survey. You won’t benefit directly by completing this survey. Findings from this study may help to influence policy makers to improve genetic services in Ireland.

If you have questions
If you have any general questions about this survey, please contact the Dr O’Byrne, Prof Sally Ann Lynch or other members of the research team at GCinIreland@gmail.com.

If taking part in this survey has raised any issues or concerns for you, please contact the research team at GCinIreland@gmail.com. They can answer your query or point you to an appropriate support or clinical services.

This information sheet and survey has been approved by the Mater Misericordiae University Hospital’s Research Ethics committee and Data Protection Office in 12/2021. If you have concerns about data collected or stored by this study, please contact Dr James O’Byrne (co-investigator, Mater) or Prof. Sally Ann Lynch (Principal investigator) at GCinIreland@gmail.com, or the Data protection Office, MMUH at dataprotection@mater.ie .

Data Protection Notice
All data will be collected and stored in line with the Data Protection Act 2018. The data will be stored on a secure encrypted device only by the research team. This data will be stored for five years in line with data protection guidelines and will be destroyed afterwards.

What's next?
Thank you for reading this information. If you would like to participate, you can find the survey by clicking "Next" below.

Genetic services survey for patients at the National Centre for Inherited Metabolic Disorders, MMUH: Information for Participants

Section 1: About you – Consent to give permission to take part in this study

Please do not use your name or the names of family members or healthcare professionals in your answers.

* 1. Are you aged 18 or over? You must be aged 18 or over to take part in this survey

- ◯ Yes
- ◯ No - survey will be exited

* 2. I consent to the data being used and stored as described in the information for participants

- ◯ Yes
- ◯ No - survey will be exited

3. I confirm that:

- I have read and understand the information
- I understand that there are minimal risks to me taking part.
- I understand that the sponsors/ investigators have such insurance as is required by law in the event of injury resulting from this research.
- I understand that all the information given will be highly confidential. My name or personal details are not collected as part of this research.
- Confidentiality of records concerning my involvement in this project will be maintained.
- When required by law, the records of this research may be reviewed by government agencies and sponsors of the research, as all anonymized data will be retained on file for a period of five years.
- I understand my participation is voluntary
- I understand that results of the study will be published in the study report, scientific journals and shared at relevant conferences.
- I have had the opportunity to contact the research team to ask questions concerning any and all aspects of the project.
- I am aware that this study has obtained ethical approval from The Mater Misericordiae University Hospital Institutional Review Board.

- ◯ Yes
- ◯ No - survey will be exited

Genetic services survey for patients at the National Centre for Inherited Metabolic Disorders, MMUH: Information for Participants

Section 2: About you

Please tell us about your experience accessing genetic services, and general information about the genetic condition.

4. Are you:

- ◯ A patient at the National Centre for Metabolic Disease
- ◯ A family member of a patient

5. What is your gender?

- ◯ Male
- ◯ Female
- ◯ Other
- ◯ Prefer not to say

6. What is your ethnicity?

- ◯ Irish
- ◯ Irish Traveller
- ◯ Any other White background
- ◯ African
- ◯ Any other Black background
- ◯ Chinese
- ◯ Any other Asian background
- ◯ Other including mixed background
- ◯ Prefer not to say

7. What age are you?

- ◯ 18-25
- ◯ 26-35
- ◯ 36-45
- ◯ 45+

8. What is the metabolic diagnosis in your family?

- ◯ Phenylketonuria
- ◯ Other

9. Pregnancy Status:

- ☐ Planning/Considering Pregnancy
- ☐ Pregnant
- ☐ Post Delivery (within last year)
- ☐ Not planning/considering pregnancy
- ☐ Prefer not to say

10. Have you/are you:

- ◯ met with a genetic counsellor in adulthood
- ◯ met with a genetic counsellor in childhood
- ◯ awaiting genetic counselling
- ◯ unaware of the option of genetic counselling
- ◯ Not sure

11. At what age were you/your family member diagnosed

- ◯ Less than 1 year old
- ◯ 1-5 years old
- ◯ 6-15 years old
- ◯ 16-25 years old
- ◯ 26-50 years old
- ◯ 50+ years old
- ◯ Still seeking a diagnosis

12. Where do you get your information about your condition/the condition in your family? (Tick all that apply)

- ☐ Family members e.g. parents
- ☐ Metabolic team
- ☐ Genetic team
- ☐ Internet
- ☐ Patient Organisation

13. Have you had genetic testing?

- ◯ Yes
- ◯ No
- ◯ Unsure

>

- ◯ Other (please specify)

[ ]

Genetic services survey for patients at the National Centre for Inherited Metabolic Disorders, MMUH: Information for Participants

14. If yes, who arranged the genetic test? (Please only add the type of healthcare professional and NOT their name)

- ◯ Genetic Consultant
- ◯ GP / family doctor
- ◯ Genetic Counsellor
- ◯ Unsure

>

- ◯ Other (if a consultant, please name the speciality e.g. Cardiology, Ophthalmology)

15. Did the same person or team who arranged the genetic test explain the test result to you / them?

- ◯ Yes
- ◯ Unsure
- ◯ No (Please tell us more in the next question)

>

- ◯ Other (Please specify)

16. If "No" and the person or team who ordered the genetic test <u>did not</u> give you the genetic test result, which type of professional gave you the results?

- ◯ Genetic consultant
- ◯ Genetic counsellor
- ◯ Non-consultant Hospital Doctor
- ◯ GP or family doctor
- ◯ Nurse

>

- ◯ Other (if consultant/specialist, please name the speciality e.g. Cardiologist or Ophthalmologist)

17. How long did you/they wait, after your/their sample was taken, for the genetic test result?

- ◯ 0-3 months
- ◯ 4-6 months
- ◯ 7-12 months
- ◯ 13-18 months
- ◯ More than 18 months
- ◯ Unsure
- ◯ Still waiting

18. How did you/they receive the genetic test result? (Tick all that apply)

- ☐ In-person appointment
- ☐ Phone call
- ☐ Letter
- ☐ E-mail
- ☐ Video call
- ☐ Parent/guardian told me/them the result
- ☐ Another family member told me/them the result
- ☐ Still waiting

>

- ☐ Other (please specify)

____

19. How would you/they have preferred to receive the genetic test result? (Tick all that apply)

- ☐ In-person appointment
- ☐ Phone call
- ☐ Letter
- ☐ E-mail
- ☐ Video call

>

- ☐ Other (please specify)

____

20. Have you been referred to Genetic services for Genetic Counselling to discuss what your diagnosis means for you and your family?

- ◯ Yes - have already attended
- ◯ Yes - waiting for first appointment
- ◯ No - have not been referred, but would like to be
- ◯ No - have not been referred and do not want to be
- ◯ Unsure

>

- ◯ Other (please specify)

[ ]

21. In which hospital were you seen for Genetic Counselling to discuss what your diagnosis means for you and your family? (Please only add the hospital name and do NOT name the healthcare professionals)

[ ]

22. Was/is your appointment for Genetic Counselling:

- ◯ Private
- ◯ Public
- ◯ Provided by the 'direct to consumer' over the counter/ internet/ mail test kit company
- ◯ Prefer not to say
- ◯ Unsure
- ◯ Not applicable – I didn't have an appointment

23. How long did you wait for the Genetic Counselling appointment? (If you are still waiting for an appointment - how long have you been waiting for?)

- ◯ 0-3 months
- ◯ 3-6 months
- ◯ 6-9 months
- ◯ 9-12 months
- ◯ 12-15 months
- ◯ 15-18 months
- ◯ 18-24 months
- ◯ More than 2 years
- ◯ Unsure
- ◯ Not applicable

24. While waiting for Genetic Counselling in Ireland I/they had: (Tick all that apply)

- ☐ genetic testing via GP
- ☐ genetic testing via public Consultant
- ☐ private genetic testing in Ireland
- ☐ private genetic testing or appointment is not something I/they could afford
- ☐ appointment to see a Genetic Consultant or Genetic Counsellor via the Cross-Border Directive or the Treatment Abroad Scheme
- ☐ genetic testing through a research study / clinical trial
- ☐ genetic testing via ‘direct to consumer’ over the counter/ internet/ mail test kits (e.g. 23 and me, Ancestry DNA, etc.)
- ☐ unsure
- ☐ none of the above

>

- ☐ private genetic testing in another country - name of country

25. What impact did being on the waiting list have on your personal life or plans? (Tick all that apply)

- ☐ delayed plans to have more children
- ☐ delayed plans to marry/settle down/commit to a relationship
- ☐ delayed plans to start a family
- ☐ delayed plans for mortgage or insurance
- ☐ changed or delayed employment
- ☐ changed or delayed education
- ☐ placed tension on relationships with partner, family members or friends
- ☐ wider impact on relative’s family planning/relationships/education/employment plans
- ☐ no impact
- ☐ not applicable

>

- ☐ Other (please specify)

26. We would like to hear more about your experience (or your family member's experience) of being on the waiting list to see a Genetic Consultant or Genetic Counsellor. Please tell us about what could have been improved, what worked well and what didn’t work well.

27. How satisfied are you/they with the overall experience of Genetic Counselling?

- Very satisfied
- Quite satisfied
- Neither satisfied nor dissatisfied
- Quite dissatisfied
- Very dissatisfied
- Not applicable

28. Is there anything else you would like to say about the experience of Genetic Counselling? Please do NOT name the healthcare professionals involved

29. Whether you have met with a genetic counsellor or not, what do you believe genetic counselling can provide? (Tick all that apply)

- [ ] Support for dealing with a genetic diagnosis in the family
- [ ] Long term psychological counselling
- [ ] Information about a genetic condition in the family
- [ ] Support for paternity testing
- [ ] Make a genetic diagnosis
- [ ] Information about how the condition is passed on (inherited) in the family
- [ ] Interpretation of genetic testing results from ‘direct to consumer’ over the counter/ internet/ mail tests (e.g. 23 and me, Ancestry DNA, etc.)
- [ ] Support for deciding whether to have a genetic test
- [ ] Support for sharing genetic information with other family members
- [ ] Support for filling in social welfare and benefit forms
- [ ] Advice about health screening and management of the condition
- [ ] Information about how a genetic condition may affect other family members
- [ ] Help linking to appropriate research or clinical trials
- [ ] Information and support about reproductive choices for a future pregnancy
- [ ] Unsure

>

- [ ] Other (please specify)

Genetic services survey for patients at the National Centre for Inherited Metabolic Disorders, MMUH: Information for Participants

Survey Finished

Thank-you very much for completing this survey. If taking part in this survey has raised any issues or concerns for you, please contact the research team at GCinIreland@gmail.com. They can answer your query or point you to appropriate supports or clinical services.

A report for policy makers, healthcare providers and the public will be published. We expect that the report will be available in 2022/23. You will be able to access the report at http://www.adelaide.ie/health-policy/publications-reports/ after it is published.
